# Neurophysiological and Neurochemical Effects of the Putative Cognitive Enhancer (*S*)-CE-123 on Mesocorticolimbic Dopamine System

**DOI:** 10.3390/biom10050779

**Published:** 2020-05-18

**Authors:** Claudia Sagheddu, Nicholas Pintori, Predrag Kalaba, Vladimir Dragačević, Gessica Piras, Jana Lubec, Nicola Simola, Maria Antonietta De Luca, Gert Lubec, Marco Pistis

**Affiliations:** 1 Department of Biomedical Sciences, Division of Neuroscience and Clinical Pharmacology, University of Cagliari, 09042 Monserrato, Italy; claudiasagheddu@unica.it (C.S.); Jose.pinto@hotmail.it (N.P.); gessica.piras@hotmail.it (G.P.); nicola.simola@unica.it (N.S.); deluca@unica.it (M.A.D.L.); 2 Department of Pharmaceutical Chemistry, Faculty of Life Sciences, University of Vienna, 1010 Vienna, Austria; predrag.kalaba@univie.ac.at (P.K.); dragacevicvladimir@gmail.com (V.D.); 3 Department of Neuroproteomics, Paracelsus Medical University, 5020 Salzburg, Austria; jana.aradska@gmail.com; 4 Neuroscience Institute, National Research Council of Italy (CNR), Section of Cagliari, 09100 Cagliari, Italy

**Keywords:** cognitive enhancer, prefrontal cortex, dopamine, dopamine transporter, modafinil, reward, psychostimulants

## Abstract

Treatments for cognitive impairments associated with neuropsychiatric disorders, such as attention deficit hyperactivity disorder or narcolepsy, aim at modulating extracellular dopamine levels in the brain. CE-123 (5-((benzhydrylsulfinyl)methyl) thiazole) is a novel modafinil analog with improved specificity and efficacy for dopamine transporter inhibition that improves cognitive and motivational processes in experimental animals. We studied the neuropharmacological and behavioral effects of the *S*-enantiomer of CE-123 ((*S*)-CE-123) and *R*-modafinil in cognitive- and reward-related brain areas of adult male rats. In vivo single unit recordings in anesthetized animals showed that (*S*)-CE-123, but not *R*-modafinil, dose-dependently (1.25 to 10 mg/kg i.v.) reduced firing of pyramidal neurons in the infralimbic/prelimbic (IL/PrL) cortex. Neither compound the affected firing activity of ventral tegmental area dopamine cells. In freely moving animals, (*S*)-CE-123 (10 mg/kg i.p.) increased extracellular dopamine levels in the IL/PrL, with different patterns when compared to *R*-modafinil (10 mg/kg i.p.); in the nucleus accumbens shell, a low and transitory increase of dopamine was observed only after (*S*)-CE-123. Neither (*S*)-CE-123 nor *R*-modafinil initiated the emission of 50-kHz ultrasonic vocalizations, a behavioral marker of positive affect and drug-mediated reward. Our data support previous reports of the procognitive effects of (*S*)-CE-123, and show a minor impact on reward-related dopaminergic areas.

## 1. Introduction

The mesocorticolimbic dopamine system is involved in processing motivation, salience, and reward, with dopamine also being a neuromodulator in brain areas that are involved in diverse cognitive processes. Reuptake and compartmentalization have a pivotal role in neurotransmission, balancing extracellular/cytosolic synaptic neurotransmitters. The dopamine transporter (DAT) is located in the presynaptic plasma membrane, where it spatially and temporally produces an accumulation of dopamine [1]. Given its multifaceted role, increasing dopamine levels by inhibition of DAT provides the opportunity to target diverse neuropsychiatric conditions. Moreover, the selective modulation of dopamine levels, particularly in the prefrontal cortex (PFC), is considered a major goal for the enhancement of mental performance in both healthy and diseased individuals [2,3]. Among the inhibitors of dopamine reuptake, cocaine produces an accumulation of dopamine in the synaptic cleft, whereas amphetamines operate as substrates for DAT to enter the presynaptic bouton, switching the activity of DAT, ultimately resulting in the efflux of dopamine. DAT-modulating drugs are approved for several medical conditions in both adults and minors. For instance, methylphenidate (MPH) and dexamphetamine are the first-choice treatment for attention deficit hyperactivity disorder (ADHD), while MPH and modafinil are prescribed for narcolepsy. Modafinil is also being clinically tested in ADHD, depression, schizophrenia, and mood disorders [4,5,6,7,8,9]. Finally, modafinil is considered as a potential therapy against substance abuse disorders at different stages [2,10], in particular against psychostimulant abuse and withdrawal [11,12,13,14,15], as well as against alcohol dependence [16].

Despite the positive effects of drugs improving cognitive functions, side effects [2] have to be taken into account, particularly when considering children and adolescents [17]. The repeated administration of MPH and modafinil is associated with behavioral sensitization and cognitive tolerance in rats [18,19]. Moreover, as these drugs target reward-related brain areas, psychostimulants are well known to carry a significant risk of abuse [20,21,22]. Such side effects of available inhibitors of dopamine reuptake are possibly due to their limited selectivity for the DAT, combined with their multitarget pharmacological profiles. The prospect of interference with healthy and impaired mental abilities (even during brain development) requires the thorough pharmacological characterization of psychostimulants as cognitive enhancers.

CE-123 5-((benzhydrylsulfinyl)methyl) thiazole) is a modafinil analog with increased affinity and selectivity for DAT. CE-123 has been shown to increase cognitive flexibility in the attentional set-shifting task paradigm [23] and to enhance memory acquisition and retrieval in a spatial hole-board task in compromised Sprague-Dawley male rats [24]. The *S* enantiomer of CE-123, (*S*)-CE-123, which was shown to be more potent at inhibiting DAT than the *R* enantiomer [23,24], reversed the effort-related effects of tetrabenazine, a blocker of vesicular monoamine transport, showing ameliorative motivational effects [23,24,25]. Camats-Perna and colleagues also demonstrated that the administration of CE-123 (racemate and *S* enantiomer) protected the consolidation of long-term social memory against interference for defined durations after learning [26].

Cognitive flexibility in humans and animals cannot be homologated. However, evidence that dopamine takes part in the control of cognitive flexibility is derived both from human and animal studies [27] showing its involvement in reversal learning, set-shifting, and task-switching. Clinical studies have shown that individuals with poor cognitive flexibility exhibit increased severity or progression in several psychiatric and neurological disorders, and deficits in cognitive flexibility are common traits in affective, anxiety, post-traumatic stress disorder and neurodegenerative disorders [28,29,30,31,32,33]. Thus, the modulation of cortical and subcortical dopamine achieved by dopaminergic drugs, such as DAT inhibitors [27], or by nonpharmacological interventions (i.e., cognitive flexibility training) [33], might ameliorate deficits in cognitive flexibility.

In this study, in vivo neurochemical, electrophysiological, and behavioral experiments were performed in adult male rats. The effect of (*S*)-CE-123 was compared to its parent compound, *R*-modafinil, which displays a more favorable pharmacokinetic profile and stronger wakefulness effects than racemic modafinil [34]. (*S*)-CE-123 and *R*-modafinil were tested at several doses in brain areas associated with cognition, such as the infralimbic/prelimbic (IL/PrL) cortex, and in areas associated with positive reinforcement induced by drugs of abuse, such as the ventral tegmental area (VTA) and the shell of the nucleus accumbens (NAc shell).

## 2. Materials and Methods 

### 2.1. Subjects

Male Sprague-Dawley rats (Envigo, Italy) weighing 250–350 g were maintained under standard conditions (temperature 22 ± 2 °C, humidity 60%, on a 12 h light/dark cycle) with ad libitum access to food and water until the experiment day. All procedures were performed in accordance with the European legislation EU Directive 2010/63 were approved by the Animal Ethics Committees of the University of Cagliari and by the Italian Ministry of Health (legislative decree 116/92). We made all efforts to minimize pain and suffering and to reduce the number of animals used.

### 2.2. Drugs and Treatments

(*S*)-CE-123 ((*S*)-*5*-((benzhydrylsulfinyl)methyl)thiazole) was synthesized from the Lubec laboratory (University of Vienna, Austria) as reported previously [23]. In short, diphenylmethanol and thiourea were reacted to yield [(diphenylmethyl)sulfanyl]methanimideamide hydrobromide. In the following step, [(diphenylmethyl)sulfanyl]methanimideamide hydrobromide was reacted under alkaline conditions with 5-(chloromethyl)thiazole hydrochloride to yield 5-((benzhydrylthio)methyl)thiazole, that was, in the subsequent synthetic step, oxidized using 30% hydrogen peroxide under acidic conditions to yield the final product, 5-((benzhydrylsulfinyl)methyl)thiazole (CE-123). CE-123 was further separated into individual enantiomers (*S*- and *R*-CE-123, respectively) using a Shimadzu 10AVP HPLC System (Shimadzu Corporation, Tokyo, Japan) equipped with Chiralpak IA semipreparative column (10 mm diameter× 20 cm length) (Chiral Technologies Europe, France). 

*R*-modafinil, (*R*)-2-(benzhydrylsulfinyl)acetamide was also synthesized in house as reported previously [35] via a procedure based on the US 7,812,193 B2 patent application.

On the day of the experiments, (*S*)-CE-123 or *R*-modafinil were freshly dissolved in DMSO 4%, TWEEN 80 5%, and physiological solution (NaCl 0.9%), and then either intravenously (i.v.) administered at cumulative doses 1.25 to 10 mg/kg/mL for electrophysiological experiments, or intraperitoneally (i.p.) injected at 1, 5, or 10 mg/kg in 3 mL for in vivo microdialysis studies and ultrasonic vocalizations (USVs) recordings. 

### 2.3. In Vivo Single-Unit Extracellular Recordings

Rats were anesthetized with 1.3 g/kg i.p. urethane. For intravenous administration of pharmacological agents, a cannula was inserted into the femoral vein. Rats were placed in a stereotaxic apparatus (Kopf, Tujunga, CA, USA) with their body temperature maintained at 37 ± 1 °C by a heating pad. The extracellular activity of neurons was recorded (bandpass filter 0.1–10,000 Hz) with glass micropipettes filled with 2% Pontamine sky blue dissolved in 0.5 M sodium acetate. Individual action potentials were isolated and amplified by means of a window discriminator (Neurolog System, Digitimer, Hertfordshire, UK) and displayed on a digital storage oscilloscope (TDS 3012, Tektronics, Marlow, UK). Experiments were sampled online and offline with Spike2 software by a computer connected to the CED1401 interface (Cambridge Electronic Design, Cambridge, UK). The single-unit activity of putative IL/PrL neurons (AP: 3.0 mm from bregma, L: ±0.8–1.0 mm from the midline) located in layers III–VI (V: 1.0–3.5 mm from the cortical surface) was recorded. The electrophysiological characteristics of the recorded cells corresponded to those attributed to pyramidal neurons, as described previously [36]. They presented “regular-spiking” or “intrinsically bursting” activity, the firing rate never exceeded 10 Hz, and the action potentials were >2 ms wide. The single-unit activity of neurons located in the ventral tegmental area (VTA, AP: −6.0 mm from bregma; L: ±0.4–0.6 mm from the midline; V: 7.0–8.0 mm from the cortical surface) was recorded extracellularly (bandpass filter 0.1–10,000 Hz). Putative dopamine neurons were isolated and identified according to published criteria [37,38], i.e., a firing rate < 10 Hz and >2.5 ms duration of the action potential. Bursts were defined as the occurrence of two spikes at an interspike interval of <80 ms, and terminated when the interspike interval exceeded 160 ms. 

### 2.4. In Vivo Brain Microdialysis: Dopamine Extracellular Levels

In vivo brain microdialysis allows us to monitor the extracellular concentration of endogenous transmitters and to estimate it as a correlate of neurotransmission. Vertical microdialysis probes were prepared with AN69 fibers (Hospal Dasco, Bologna, Italy), as previously described [39,40]. In order to evaluate dopamine extracellular levels by in vivo brain microdialysis, rats were anaesthetized with Equithesin (3 mL/kg i.p.; chloral hydrate 2.1 g, sodium pentobarbital 0.46 g, MgSO_4_ 1.06 g, propylene glycol 21.4 mL, ethanol (90%) 5.7 mL, H_2_O 3 mL) and were implanted with microdialysis probes. The microdialysis probe was implanted in the NAc shell (AP: +2.2 mm, L: +1.0 mm from bregma, V: −7.8 mm from dura; 1.5 mm dialyzing portion) or in the IL/PrL cortex (AP: +3.7 mm, L: +0.8 mm from bregma, V: −5.0 mm from dura; 3 mm dialyzing portion) according to the rat brain atlas [41]. The day of the experiment, probes were perfused with Ringer’s solution (147 mM NaCl, 4 mM KCl, 2.2 mM CaCl_2_) at a constant rate of 1 µL/min. Dialysate samples (20 µL) were injected into an HPLC equipped with a reverse-phase column (C8 3.5 um, Waters, Sesto San Giovanni (MI), Italy USA) and a coulometric detector (ESA, Coulochem II) to quantify dopamine. The first electrode of the detector was set at +130 mV (oxidation) and the second at −175 mV (reduction). The composition of the mobile phase was 50 mM NaH_2_PO_4_, 0.1 mM Na_2_-EDTA, 0.5 mM n-octyl sodium sulfate, 15% (*v*/*v*) methanol, pH 5.5. The sensitivity of the assay for dopamine was 5 fmol/sample. After the evaluation of basal levels, estimated as the mean of three consecutive samples whose values did not differ more than ±10%, animals received an i.p. injection with (*S*)-CE-123, *R*-modafinil or vehicle, and the dopamine extracellular levels were monitored up to 3 h after the injection. At the end of the experiments, localization of probes for microdialysis was shown by histological examination

### 2.5. Recording of 50-kHz USVs

USV emissions were recorded from individual rats during microdialysis experiments by means of ultrasonic microphones (CM16/CMPA, Avisoft, Berlin, Germany) connected to an ultrasound recording device (UltraSoundGate 116 Hb, Avisoft, Berlin, Germany), as previously described [42]. Intensity gain was kept at a constant level during the recordings. USV emissions were recorded for 3 h after the administration of (*S*)-CE-123, *R*-modafinil or vehicle, starting immediately after injection. 

### 2.6. Data Analysis

Sample sizes were determined based both on our previous experience in the experimental protocols and the expected effect size. Experiments were excluded from analysis if incomplete or following histological verification of the recording site (for electrophysiology experiments) or localization of microdialysis probe. To reduce animal pain and suffering, we minimized the number of animals used for each experimental set to the minimum required for statistical analysis validation.

For all experiments, statistical analyses were performed using either Statistica (StatSoft, Tulsa, OK, USA), or Prism (GraphPad, La Jolla, CA, USA) software. 

For dose-curve electrophysiological experiments, drug-induced changes in firing activity were calculated by averaging the effects of the drugs for the 2 min period following drug administration. Statistical significance was then assessed using one-way ANOVA for repeated measures, followed by Dunnett’s correction for multiple comparisons. For single-dose in vivo microdialysis experiments, data were analyzed by two-way ANOVA (treatment × time) for repeated measures followed by Tukey’s post-hoc test. Data analysis of USVs was performed as previously described [42]. Briefly, USV recordings were converted into spectrograms by means of the software SASLab Pro 4.52 with the following settings: 512 Fast Fourier Transform (FFT)-length, Hamming window, and 75% overlap frame set-up. Spectrograms were manually processed to remove background noise and signals that could not be unambiguously classified as USVs. Thereafter, the total number of 50-kHz USVs isolated in each spectrogram was automatically counted by means of SASLab Pro 4.52. The total numbers of 50-kHz USVs emitted after administration of (*S*)-CE-123, *R*-modafinil, or vehicle were analyzed by two-way ANOVA (treatment × time). All the numerical data are expressed as means ± S.E.M. Significance level was set at *p* < 0.05 for each analysis.

## 3. Results

### 3.1. Effects of (S)-CE-123 and R-Modafinil on In Vivo Firing Rate of Putative Pyramidal Neurons from the IL/PrL Cortex

**Figure 1:**
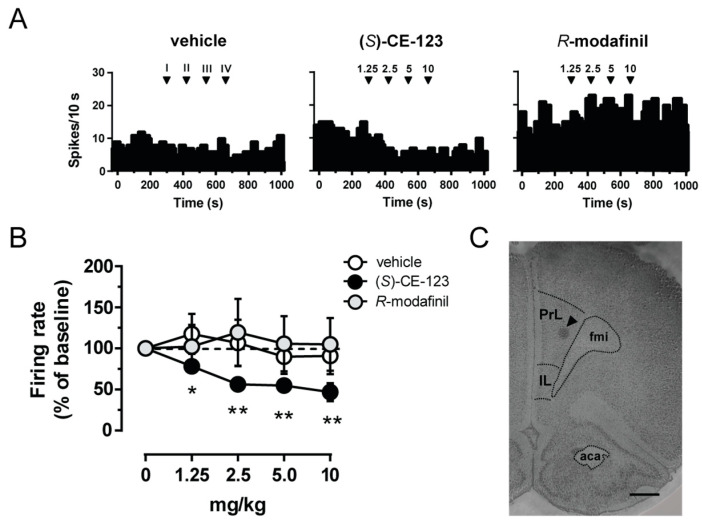
Effect of (*S*)-CE-123 and *R*-modafinil on in vivo electrical activity of putative pyramidal cells. Representative firing rate histograms of putative pyramidal neurons (**A**) from rats that received cumulative intravenous injections of vehicle (left), (*S*)-CE-123 (10 mg/kg; middle) or *R*-modafinil (10 mg/kg; right). Arrows indicate the time of injections and number of the dose (mg/kg). (*S*)-CE-123 reduced firing frequency (**B**) in a dose-dependent manner (vehicle n = 5; *R*-modafinil n = 7, (*S*)-CE-123 n = 7). Symbols and bars represent means ± SEM, * *p* < 0.05, ** *p* < 0.01, RM one-way ANOVA and Dunnett’s test. (**C**) Histological brain section showing the recording site in the prelimbic/infralimbic cortex. The black triangle indicates the pontamine sky blue dye. Abbreviations: PrL, prelimbic cortex; IL, infralimbic cortex; aca, anterior commissure, anterior part; fmi, forceps minor of the corpus callosum. Scale bar 1 mm.

We examined the in vivo electrophysiological effects of (*S*)-CE-123 and *R*-modafinil on putative pyramidal neurons recorded from the IL/PrL cortex of anesthetized rats (Figure 1C). Cumulative i.v. administration (1.25–10 mg/kg) of (*S*)-CE-123 produced a dose-dependent decrease in the cell firing rate (example rate histogram in Figure 1A,B; one-way ANOVA for repeated measures, F_(6,24)_ = 19.26, *p* = 0.0009, Dunnett’s multiple comparisons test). By contrast, *R*-modafinil at the same doses did not produce a significant effect on the neuron firing rate (example rate histogram in Figure 1A,B; one-way ANOVA for repeated measures F_(6,24)_ = 0.231, *p* = 0.684). As expected, vehicle administration was also ineffective (example rate histogram in Figure 1A,B; one-way ANOVA for repeated measures, F_(4,16)_ = 0.928, *p* = 0.414). 

### 3.2. Effects of (S)-CE-123 and R-Modafinil on In Vivo Firing Activity of Putative VTA Dopamine Neurons

**Figure 2:**
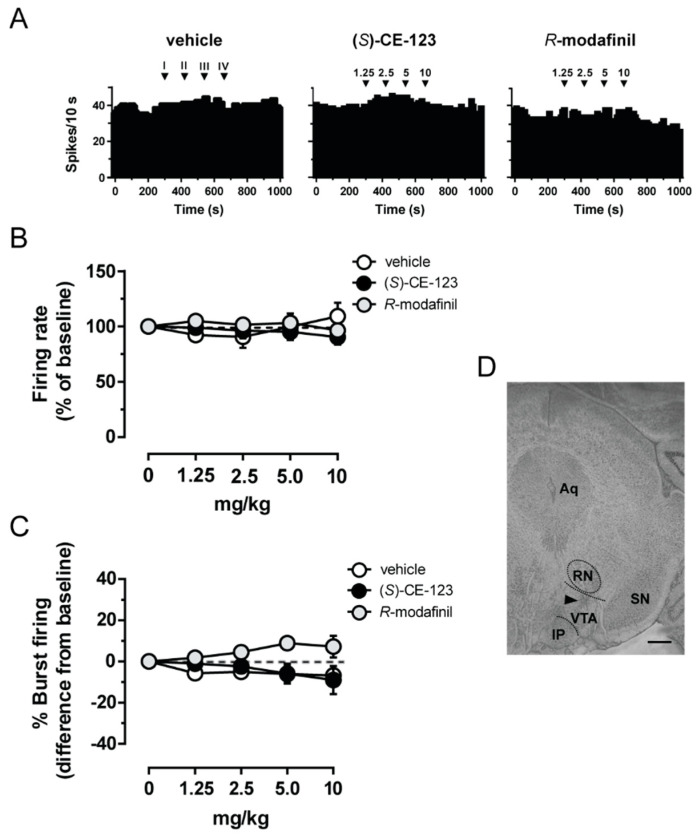
Effect of (*S*)-CE-123 and *R*-modafinil on in vivo electrical activity of putative dopamine cells. Representative firing rate histograms of putative ventral tegmental area (VTA) dopamine neurons (**A**) from rats that received cumulative intravenous injections of vehicle (left), (*S*)-CE-123 (10 mg/kg; middle) or *R*-modafinil (10 mg/kg; right). Arrows indicate the time of injections and number of the dose (mg/kg). *R*-modafinil and (*S*)-CE-123 did not change firing frequency (**B**) or bursting activity (**C**) of putative VTA dopamine neurons (vehicle n = 5; *R*-modafinil n = 6, (*S*)-CE-123 n = 7). Symbols and bars represent means ± SEM, RM one-way ANOVA. (**D**) Histological brain section showing the recording site in the VTA. The black triangle indicates the pontamine sky blue dye. Abbreviations: Aq, aqueduct; RN, red nucleus; IP, interpeduncular nucleus; SN, substantia nigra; VTA, ventral tegmental area. Scale bar, 0.5 mm.

We next studied the effects of (*S*)-CE-123 and *R*-modafinil (cumulative doses: 1.25–10 mg/kg i.v.) on the firing activity of putative VTA dopamine neurons (Figure 2D). Neither (*S*)-CE-123 nor *R*-modafinil (example rate histograms in Figure 2A) affected the firing frequency (Figure 2B; one-way ANOVA for repeated measures, F_(6,24)_ = 1.304, *p* = 0.303 and F_(5,20)_ = 1.343, *p* = 0.305, respectively). Similarly, neither (*S*)-CE-123 (Figure 2C; one-way ANOVA for repeated measures, F_(6,24)_ = 1.671 *p* = 0.242) nor *R*-modafinil (Figure 2C; one-way ANOVA for repeated measures, F_(5,20)_ = 2.810, *p* = 0.133) changed the burst firing of putative VTA dopamine cell. Finally, the vehicle (example rate histogram in Figure 2A) was ineffective with regard to both the firing rate (Figure 2B; one-way ANOVA for repeated measures, F_(4,16)_ = 1.682, *p* = 0.254) and burst firing (Figure 2C one-way ANOVA for repeated measures, F_(4,16)_ = 2.612, *p* = 0.171).

### 3.3. Effect of (S)-CE-123 and R-Modafinil on Dopamine Transmission in the IL/PrL Cortex and in the NAc Shell

**Figure 3:**
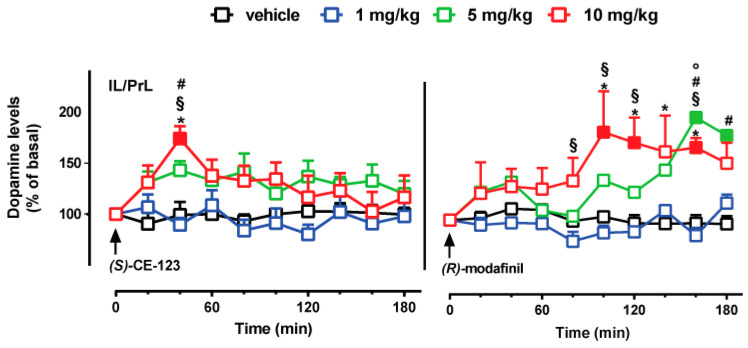
Effect of (*S*)-CE-123 and *R*-modafinil on dopamine transmission in the IL/PrL cortex. Graphs showing the effects of (*S*)-CE-123 (left) or *R*-modafinil (right) on dopamine levels in the IL/PrL cortex. The arrow indicates i.p. injection of (*S*)-CE-123 or *R*-modafinil at the dose of 1 mg/kg (blue), 5 mg/kg (green), 10 mg/kg (red) or vehicle (black). Results are presented as mean ± SEM of change in dopamine extracellular levels expressed as the percentage of basal values. Solid symbol: *p* < 0.05 with respect to basal values; * *p* < 0.05 (*S*)-CE-123 10 mg/kg vs. vehicle; § *p* < 0.05 (*S*)-CE-123 10 mg/kg vs. (*S*)-CE-123 1 mg/kg dose; # *p* < 0.05 (*S*)-CE-123 5 mg/kg vs. veh; ° *p* < 0.05 *R*-modafinil 10 mg/kg vs. (*S*)-CE-123 10 mg/kg ((*S*)-CE-123 n = 21; *R*-modafinil n = 12). Three-way ANOVA, Tukey’s post hoc.

**Figure 4:**
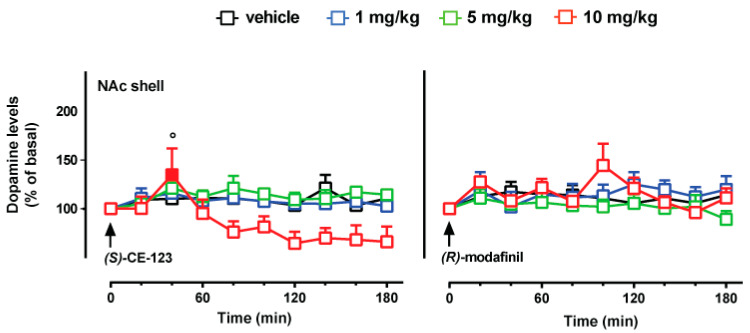
Effect of (*S*)-CE-123 and *R*-modafinil on dopamine transmission in the NAc shell. Graphs showing the effects of (*S*)-CE-123 (left) or *R*-modafinil (right) on dopamine levels in the shell of the NAc. The arrow indicates i.p. injection of (*S*)-CE-123 or *R*-modafinil at the dose of 1 mg/kg (blue), 5 mg/kg (green), 10 mg/kg (red) or vehicle (black). Results are presented as mean ± SEM of change in dopamine extracellular levels expressed as the percentage of basal values. Solid symbol: *p* < 0.05 with respect to basal values; ° *p* < 0.05 (*S*)-CE-123 10 mg/kg vs. *R*-modafinil 10 mg/kg ((*S*)-CE-123 n = 21; *R*-modafinil n = 15). Three-way ANOVA, Tukey’s post hoc.

In these experiments, we studied the effect of three doses of (*S*)-CE-123 and *R*-modafinil (1, 5 and 10 mg/kg i.p.) on extracellular dopamine levels in the IL/PrL cortex (Figure 3) and in the NAc shell (Figure 4). 

Considering IL/PrL cortex, a three-way ANOVA showed a main effect of dose [F_(3,25)_ = 8.09; *p* = 0.000062], of time [F_(9,225)_ = 4.21; *p* = 0.00005], a significant treatment × time interaction [F_(9,225)_ = 4.42; *p* = 0.000025], dose × time interaction [F_(27,225)_ = 2.87; *p* = 0.018], and treatment × dose × time interaction [F_(27,225)_ = 2.57; *p* = 0.00008]. Tukey’s post hoc tests showed a significant increase of dialysate dopamine at 40 min after the administration of (*S*)-CE-123 (10 mg/kg i.p.), as compared to basal value, to vehicle and to the lower dose of (*S*)-CE-123 (1 mg/kg i.p.). A Tukey’s post hoc tests also showed a significant increase of dopamine at 100, 120, and 160 min after administration of the higher dose of *R*-modafinil (10 mg/kg i.p.) with respect to basal value, from 100 to 160 min concerning the vehicle group, but also at 80, 100, 120, and 160 min with respect to the lower dose of *R*-modafinil (1 mg/kg i.p.). Moreover, Tukey post hoc tests revealed significant differences at 160 and 180 min after the administration of 5 mg/kg i.p. of *R*-modafinil with respect to both basal value and vehicle-treated animals, and significant differences at 160 min between animals treated with (*S*)-CE-123 (10 mg/kg i.p.) or *R*-modafinil (10 mg/kg i.p.). 

Considering the NAc shell, three-way ANOVA showed a main effect of time [F_(9,252)_ = 2.98; *p* = 0.0021], and a significant dose × time interaction [F_(27,252)_ = 1.54; *p* = 0.045]. Tukey’s post hoc tests showed a significant increase of dialysate dopamine at 40 min after the administration of (*S*)-CE-123 (10 mg/kg i.p.), to the basal value; it also showed significant differences at 40 min between rats treated with (*S*)-CE-123 (10 mg/kg i.p.) and *R*-modafinil (10 mg/kg i.p.).

### 3.4. Effect of (S)-CE-123 and R-Modafinil on the Emission of 50-kHz USVs

**Figure 5:**
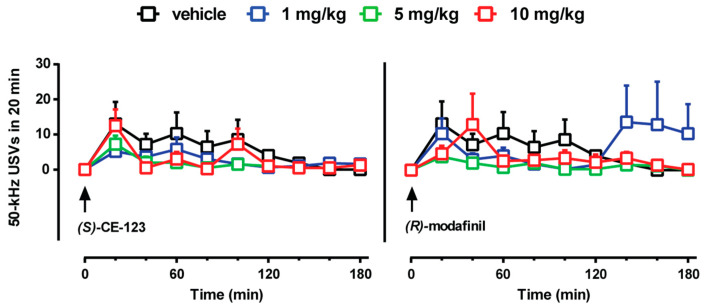
Effect of (*S*)-CE-123 and *R*-modafinil on the emission of 50-kHz ultrasonic vocalizations. Graphs showing the effects of (*S*)-CE-123 (left) or *R*-modafinil (right) on the emission of 50-kHz USVs. The arrow indicates i.p. injection of (*S*)-CE-123 or *R*-modafinil at the dose of 1 mg/kg (blue), 5 mg/kg (green), 10 mg/kg (red) or vehicle (black). Results are presented as means ± SEM of the absolute numbers of 50-kHz USVs emitted. (*S*)-CE-123 or *R*-modafinil did not increase the numbers of 50-kHz ultrasonic calls emitted, as compared to vehicle. USVs = ultrasonic vocalizations, vehicle n = 7; (*S*)-CE-123 n = 13; *R*-modafinil n = 13. Two-way ANOVA.

We tested the effect of 1, 5, and 10 mg/kg (*S*)-CE-123 and *R*-modafinil on the emission of 50-kHz USVs, a behavioral marker of positive affect and drug-induced reward, in a subset of freely moving rats during brain microdialysis. Acute administration of (*S*)-CE-123 (1–10 mg/kg, i.p.) did not increase the numbers of 50-kHz USVs emitted, as compared to vehicle administration (Figure 5, left). Two-way ANOVA revealed no significant effect of treatment (F_(3,16)_ = 0.76, *p* = 0.54) and no significant treatment × time interaction (F_(24,128)_ = 0.77, *p* = 0.76), although a significant effect of time was observed (F_(8,128)_ = 4.92, *p* = 0.00002). Similarly, acute administration of *R*-modafinil (1–10 mg/kg, i.p.) did not elevate the numbers of 50-kHz USVs emitted, as compared to vehicle administration (Figure 5, right). Two way ANOVA revealed no significant effect of treatment (F_(3,16)_ = 0.79, *p* = 0.51) and time (F_(8,128)_ = 1.06, *p* = 0.39), and no significant treatment × time interaction (F_(24,128)_ = 1.28, *p* = 0.18). 

## 4. Discussion

Dopaminergic neuromodulation in frontal lobes and in subcortical brain areas is involved in processing cognitive skills, such as attention and associative learning [43,44], and motivation [45], respectively. In spite of the common origin from the VTA, mesocorticolimbic dopamine projections to the NAc and to the IL/PrL cortex are differently involved in behavioral tasks, and differently influenced by drugs [46]. Successful cognitive enhancement with a reduced risk potential for addiction should be then based on the selective modulation of the mesocortical dopamine pathway, such that cognition-enhancing doses of dopamine modulators preferentially elevate extracellular dopamine within the IL/PrL cortex [3,47]. Interestingly, the administration of modafinil in humans has been associated with increased prefrontal cortical activation [4]. Our experiments in the rat IL/PrL cortex showed that pyramidal neuron firing frequency was not affected by *R*-modafinil (cumulative 10 mg/kg i.v.) in anesthetized rats, while (*S*)-CE-123 produced a dose-dependent decrease. 

*R*-modafinil (5 and 10 mg/kg i.p.) increased extracellular dopamine levels with a delayed and protracted effect, while (*S*)-CE-123 (10 mg/kg) rapidly stimulated dopamine transmission. This is consistent with a microdialysis study showing that 128 mg/kg modafinil enhanced dopamine levels in prefrontal cortex, with the highest dopamine levels occurring within 2–3 h [48]. 

It is well established that cognitive and executive functions are modulated by dopamine, depending on cellular localization of postsynaptic receptors and their downstream signaling, which can be excitatory or inhibitory [49,50]. D1- and D2-like receptors are expressed in both pyramidal cells and inhibitory interneurons [51], accounting for various effects of dopamine. D1-like dopamine receptors, highly expressed in GABAergic prefrontal interneurons [51], were demonstrated to mediate the signal to noise ratio in an inverted-U dose-response [52], suggesting that neuromodulation requires highly regulated extracellular dopamine levels. Accordingly, psychostimulant drugs targeting the DAT are promising as cognitive enhancers, but have also been demonstrated to produce behavioral effects associated with abuse liability in experimental animals [53] and humans [54]. Hence, we also tested (*S*)-CE-123 and *R*-modafinil for neuropharmacological effects in rat brain areas related with reinforcement. The selective increase of dopamine neurotransmission in the NAc shell plays a pivotal role in processing reward and motivated behaviors following intake of drugs of abuse [45,55]. Our microdialysis experiments showed that i.p. administration of (*S*)-CE-123 does not increase dopamine levels (except for a low and transitory increase at 40 min at the 10 mg/kg dose). In contrast, at 10 mg/kg a downward trend of dopamine levels was observed from 80 to 180 min following administration. This lack of stimulation of dopamine transmission in the NAc shell is consistent with what observed at the dose of 24 mg/kg ip [25], although a recent study reported that (*S*)-CE-123 at doses of 10 and 100 mg/kg increased dopamine extracellular levels in the NAc shell of mice [26]. Differences in the utilized doses of (*S*)-CE-123 in the metabolic pathway of species (mice vs. rats), together with the specificity of the brain area implanted, might be the reason of the lack of increase observed in the present study. Similar to (*S*)-CE-123, none of the tested doses of *R*-modafinil stimulated dopamine transmission in the NAc shell. This observation is inconsistent with previous findings, since both Mereu et al. (2017) [56] and Keighron et al. (2019) [57] reported that modafinil increases dopamine levels in NAc shell. However, discrepancies between our results and previous reports might be due to differences in dose regimen, as well as in the route of administration. Indeed, similarly to Keighron et al. (2019) [57], preliminary experiments performed in our laboratories revealed increased dopamine levels in the NAc shell after intravenous administration of 30 mg/kg of *R*-modafinil.

Differences between the mechanisms of action of (S)-CE-123 and the parent compound modafinil might explain our results. Multiple neurotransmitter systems have been implicated in modafinil activity [10,58], as several studies have reported significant occupancy of NET, beside activity at DAT [59]. Hence, in cells expressing human DAT, R-modafinil inhibits DAT (IC_50_ 6.4–13 μM) and NET activity (IC_50_ 35.6–182 μM), albeit with lower potency in the case of the latter [34,60,61]. On the other hand, (S)-CE-123 shows negligible activity at NET or SERT, and blocks DAT (IC_50_ = 4.6 μM) without acting as a substrate [24]. Moreover, a pharmacokinetic analysis indicated that (S)-CE-123 undergoes a more rapid brain uptake and reaches higher brain levels than those of R-modafinil [24].

Our in vivo electrophysiological recordings showed that VTA dopamine cell firing frequency and bursting activity were not substantially affected by the intravenous administration of either (*S*)-CE-123 or *R*-modafinil up to the 10 mg/kg cumulative dose. Analogous doses of well-known drugs of abuse targeting DAT (such as cocaine and amphetamine) have been consistently shown to reduce dopamine neuron firing [62], possibly by dendritic dopamine release and feedback inhibition through somatodendritic D2 autoreceptors [63], and to increase extracellular dopamine in the rat shell of the NAc [64]. In agreement with this, MRI data highlighted that modafinil robustly activates the fronto-cortical areas involved in higher cognitive functions and a network of pro-arousing areas [65], though reward-associated brain areas are not functionally modulated by modafinil, even at higher doses [66,67]. 

As a behavioral marker of positive affect and drug-induced reward in rats [68], we evaluated the emission of 50-kHz USVs after the administration of (*S*)-CE-123 and *R*-modafinil. Indeed, the activation of dopamine receptors in the NAc shell has been shown to be a critical mechanism that initiates the emission of 50-kHz USVs [69,70]. Moreover, rats treated with psychostimulants targeting DAT or other drugs of abuse may emit high numbers of 50-kHz USVs, so that such calling behavior is considered a marker for the rewarding and motivational properties of drugs [71,72,73,74,75,76]. Noteworthy, in our study is the fact that different doses of acutely administered (*S*)-CE-123 and *R*-modafinil (1–10 mg/kg, i.p.) did not alter 50-kHz USV emissions in adult male rats, consistent with the weak effects that both drugs elicited on dopamine release in the NAc shell. The lack of effect of (*S*)-CE-123 on 50-kHz USV emissions marks a clear difference with other DAT-inhibiting compounds with abuse potential.

## 5. Conclusions

In conclusion, the differential effects of (*S*)-CE-123 and *R*-modafinil in the mesocorticolimbic system are in accordance with published studies showing the behavioral and neurochemical peculiarities of analogue compounds, despite their chemical similarities to the drug from which they were derived [57]. The present study suggests that low doses of (*S*)-CE-123 are effective in frontal brain areas associated with cognition, with minimal effect in brain areas typically related to the rewarding properties of drugs of abuse.
